# Supplementary material for: A Novel Phenazine Analog, CPUL1, Suppresses Autophagic Flux and Proliferation in Hepatocellular Carcinoma: Insight from Integrated Transcriptomic and Metabolomic Analysis
Source: Cancers (Basel). 2023 Mar 5;15(5):1607. doi: 10.3390/cancers15051607 (PMC10001020; doi:10.3390/cancers15051607)
Supplement: Supplementary file 1 [file cancers-15-01607-s001.zip › Supplementary Materials.pdf]

**Supplementary Materials**

# A novel phenazine analog, CPUL1, suppresses autophagic flux and proliferation in hepatocellular carcinoma: insight from integrated transcriptomic and metabolomic analysis

Jiaqin Chen[1,†], Dong Feng[2,†], Yuanyuan Lu[1,†], Yanjun Zhang[1], Hanxiang Jiang[1], Man Yuan[3], Yifan Xu[3], Jianjun Zou[4, 5], Yubing Zhu[4, 5], Jingjing Zhang[4, 5], Chun Ge[4, 5,*], Ying Wang[1,*]

[1]*School of Life Science and Technology, China Pharmaceutical University, Nanjing 211198, China*

[2]*Nanjing Southern Pharmaceutical Technology Co., Ltd., Nanjing 211100, China*

[3]*Department of Clinical Pharmacy, School of Basic Medicine & Clinical Pharmacy, China Pharmaceutical University, Nanjing 210009, China*

[4]*Department of Pharmacy, Nanjing First Hospital, Nanjing Medical University, Nanjing 210006, China*

[5]*Department of Clinical Pharmacology, Nanjing First Hospital, Nanjing Medical University, Nanjing 210006, China*

[*]Correspondence:

Chun Ge (gechun901020@163.com), Ying Wang (waying@cpu.edu.cn)

[†]These authors have contributed equally to this work.

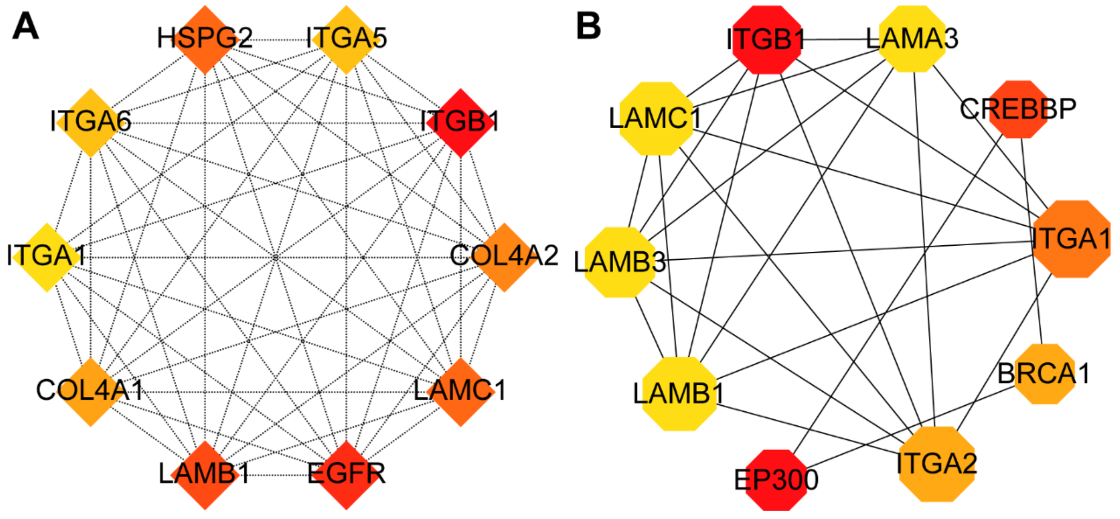


**Figure S1 The top-10 hub gene network.** This network was calculated using the degree algorithm of the Cytohubba plug-in. (A) The hub gene expression in 6h- CPUL1-treated and control. (B) The hub gene expression in 48h- CPUL1-treated and control.

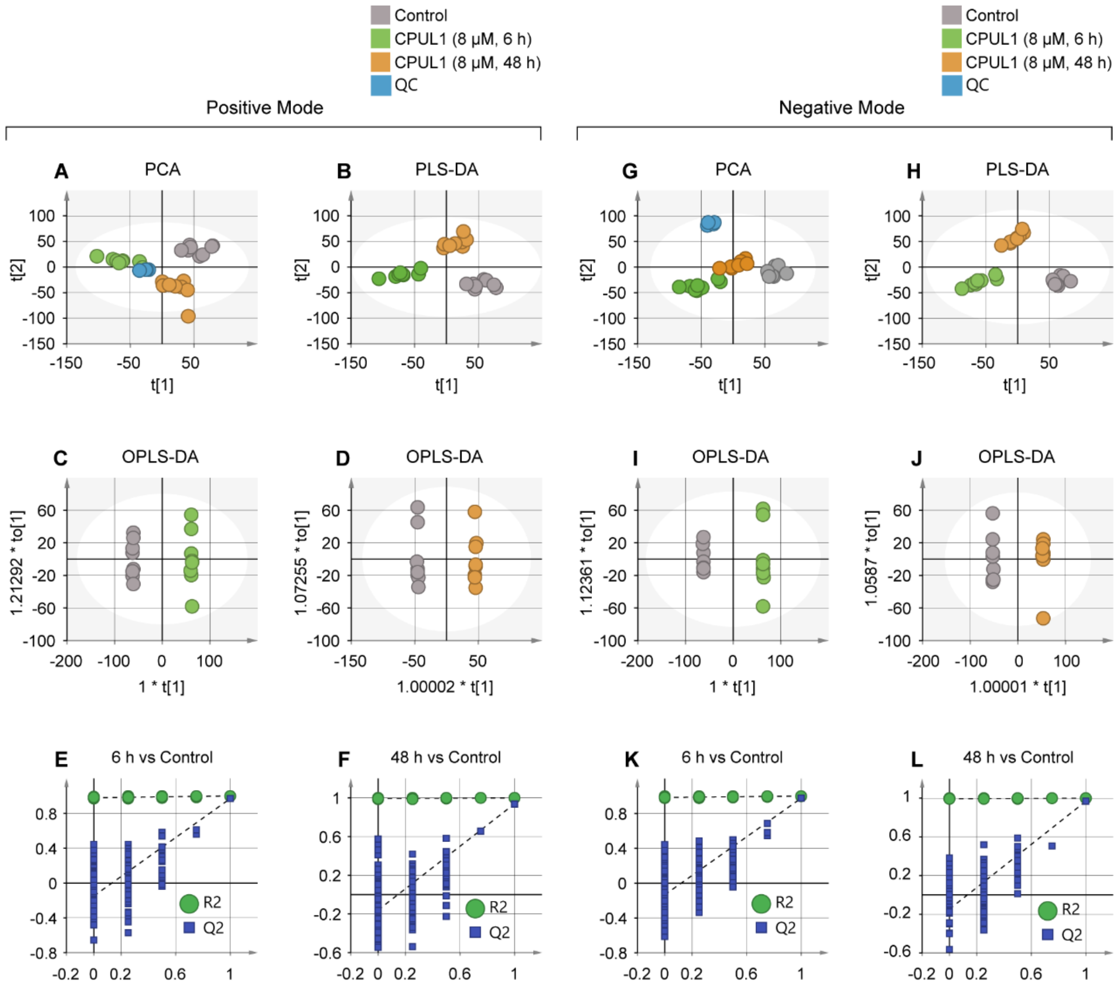


**Figure S2 Metabolomic analysis of CPUL1-treated BEL-7402 cells.** (A) Score plot from the PCA model classifying control, 6h-CPUL1-treated, 48h-CPUL1-treated, and QC groups in positive mode. (B) PLS-DA score plot of control and treated cells in positive mode. (C) OPLS-DA analysis between the control and the 6h-CPUL1-treated groups showed a good separation trend in positive mode. (D) OPLS-DA analysis between the control and the 48h-CPUL1-treated groups showed a good separation trend in positive mode. (E) Permutations plot analysis between the control and the 6h-CPUL1-treated groups showed the intersection of the Q2 regression line and the y-axis was less than zero in positive mode, indicating that the model was valid. (F) Permutations plot analysis between the control and the 48h-CPUL1-treated groups showed the intersection of the Q2 regression line and the y-axis was less than zero in

positive mode, indicating that the model was valid. (G) Score plot from the PCA model classifying control, 6h-CPUL1-treated, 48h-CPUL1-treated, and QC groups in negative mode. (H) PLS-DA score plot of control and treated cells in negative mode. (I) OPLS-DA analysis between the control and the 6h-CPUL1-treated groups showed a good separation trend in negative mode. (J) OPLS-DA analysis between the control and the 48h-CPUL1-treated groups showed a good separation trend in negative mode. (K) Permutations plot analysis between the control and the 6h-CPUL1-treated groups showed the intersection of the Q2 regression line and the y-axis was less than zero in negative mode, indicating that the model was valid. (L) Permutations plot analysis between the control and the 48h-CPUL1-treated groups showed the intersection of the Q2 regression line and the y-axis was less than zero in negative mode, indicating that the model was valid.

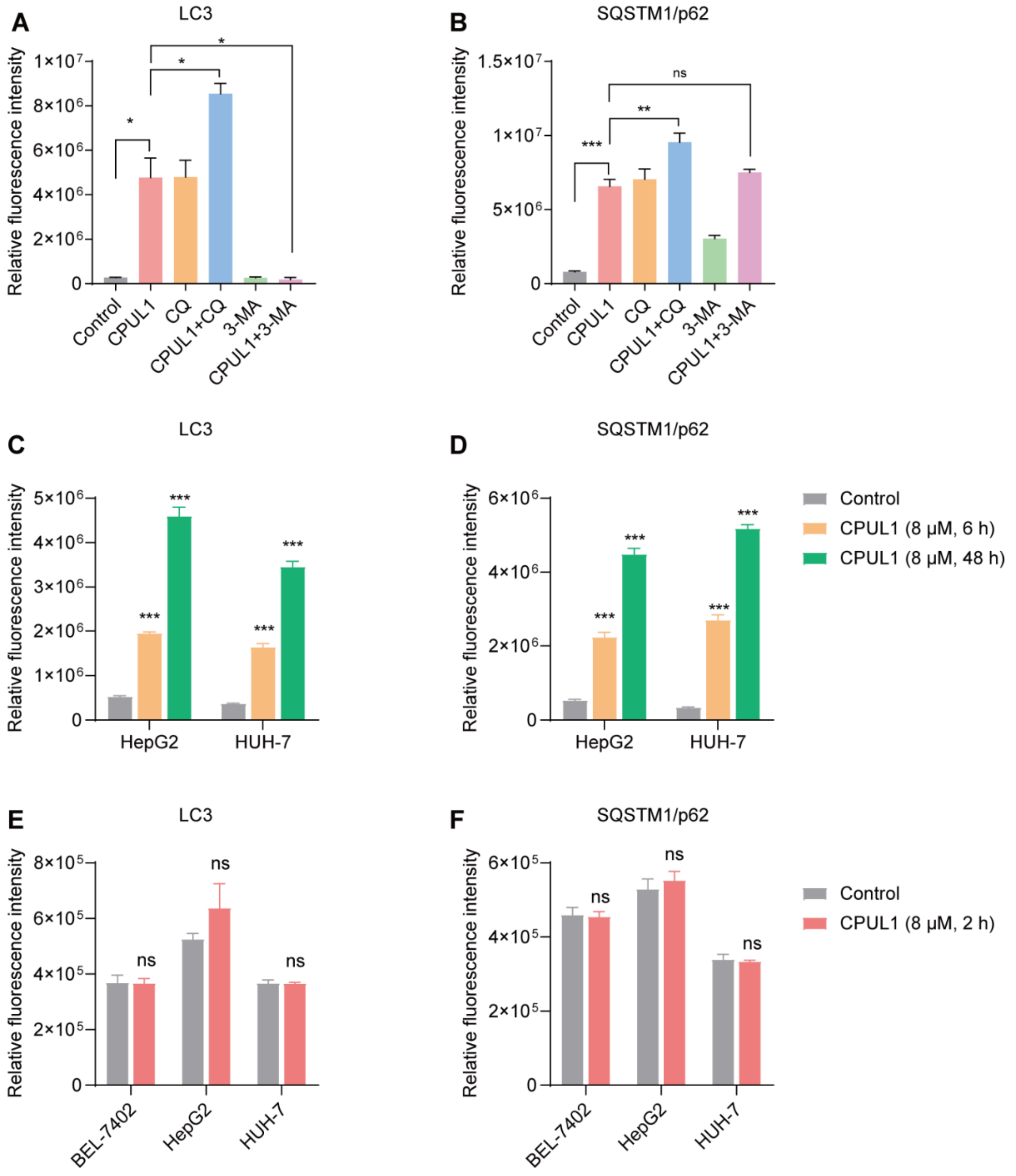


**Figure S3 Quantitative analysis of fluorescent intensity of p62 and LC3 was performed to confirm the protein changes.** (A, B) Fluorescence quantification of LC3 and p62 in BEL-7402 cells treated with CPUL1 (8 μM) and 3-MA (2 mM) or CQ (20 μM) for 48 h. (C, D) Fluorescence quantification of LC3 and p62 in HepG2 and HUH-7 cells treated with CPUL1 (8 μM) for 6 h or 48 h. (E, F) Fluorescence quanti-fication of LC3 and p62 in BEL-7402, HepG2 and HUH-7 cells treated with CPUL1 (8 μM) for 2 h.

**Table S1**. The summary of RNA sequencing data.

| Samples | Raw reads (Mb) | Clean reads (Mb) | Q30% | GC% | Mapping rate |
|---|---|---|---|---|---|
| BEL-7402-1 | 40.79 | 40.44 | 94.69 | 52.60 | 92.22% |
| BEL-7402-2 | 36.59 | 36.30 | 94.42 | 52.65 | 93.03% |
| BEL-7402-3 | 41.87 | 41.56 | 94.49 | 52.78 | 92.71% |
| BEL-7402-6h-1 | 44.12 | 43.63 | 94.81 | 52.94 | 93.22% |
| BEL-7402-6h-2 | 41.23 | 40.90 | 94.52 | 53.26 | 93.01% |
| BEL-7402-6h-3 | 43.22 | 42.80 | 94.80 | 52.83 | 93.03% |
| BEL-7402-48h-1 | 47.02 | 46.71 | 94.83 | 53.29 | 93.37% |
| BEL-7402-48h-2 | 49.57 | 49.24 | 94.70 | 53.24 | 93.23% |
| BEL-7402-48h-3 | 38.61 | 38.29 | 95.01 | 53.31 | 93.46% |
