# Supplementary figures and images for: A Novel Phenazine Analog, CPUL1, Suppresses Autophagic Flux and Proliferation in Hepatocellular Carcinoma: Insight from Integrated Transcriptomic and Metabolomic Analysis
Source: Cancers (Basel). 2023 Mar 5;15(5):1607. doi: 10.3390/cancers15051607 (PMC10001020; doi:10.3390/cancers15051607)

# mTOR

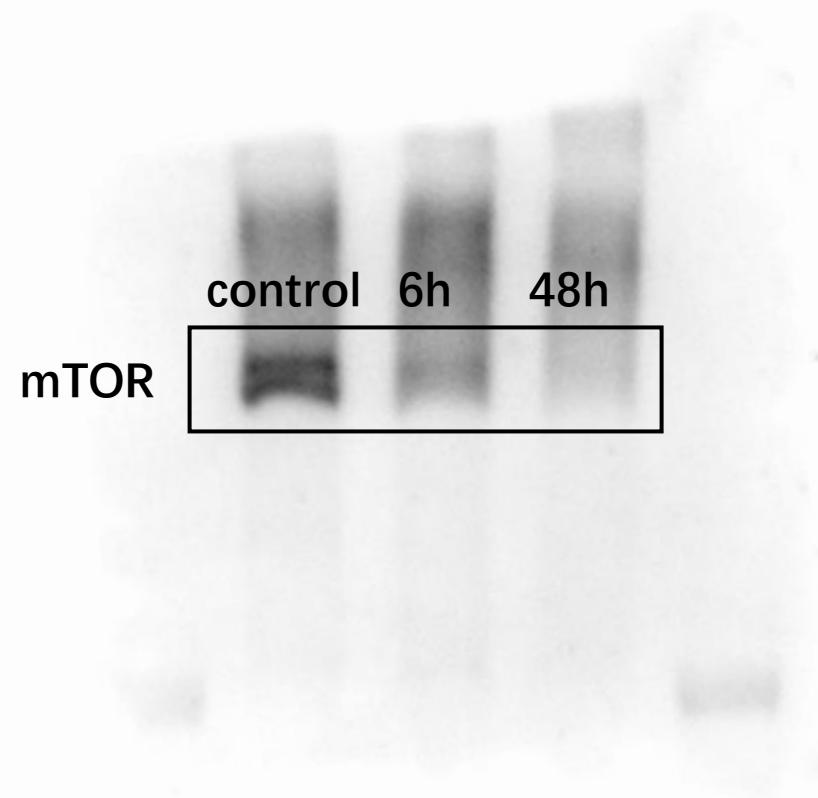

# P-mTOR

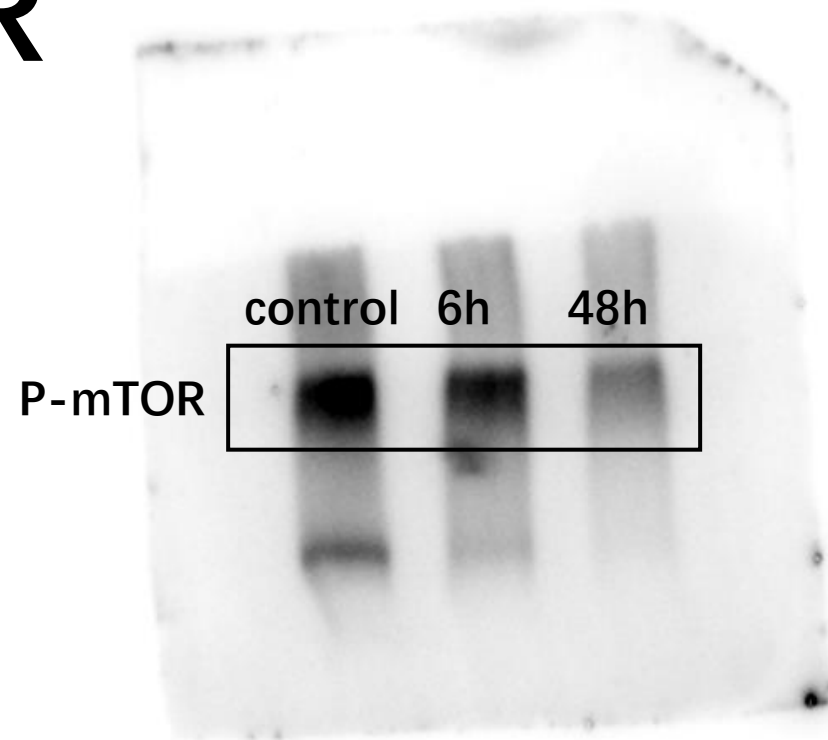

# p62

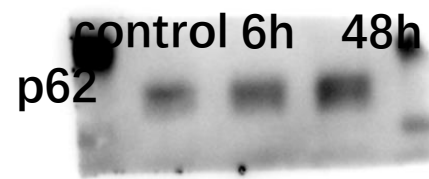

# LC3

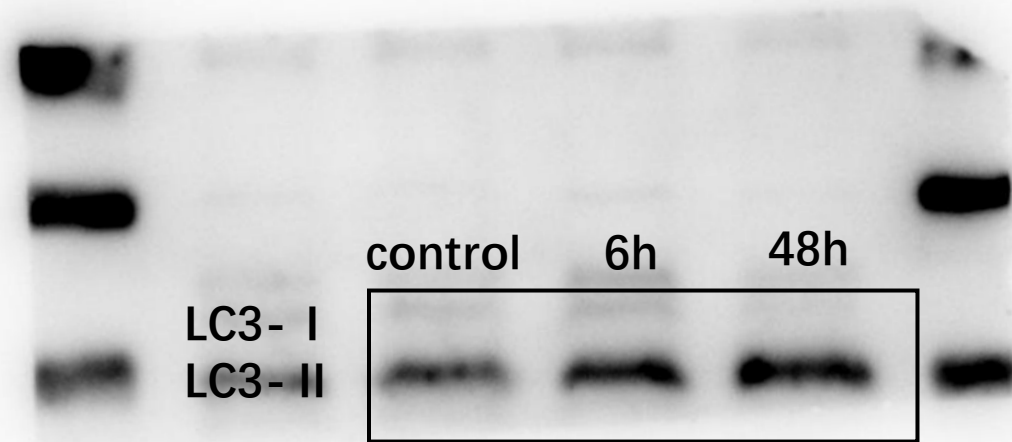

# ATG12

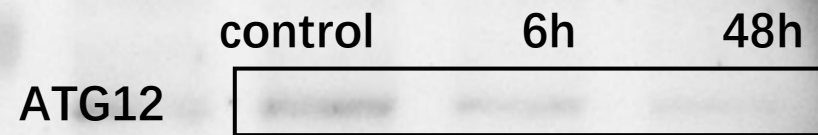

# ATG5

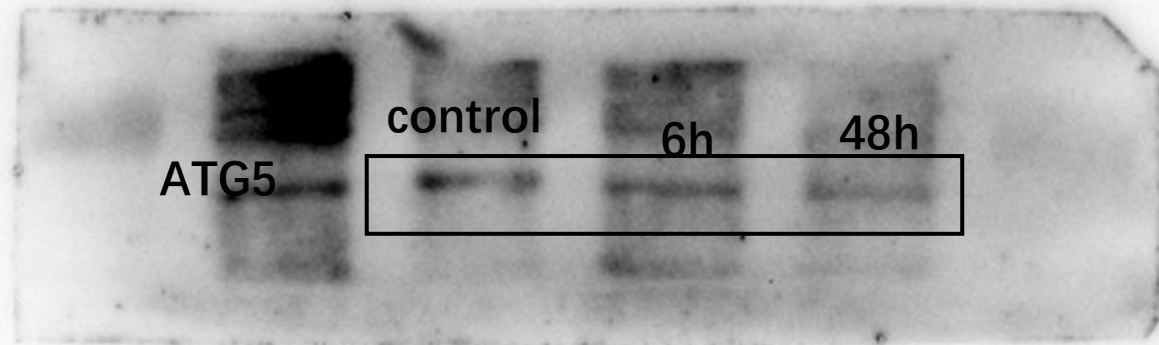

# Beclin1

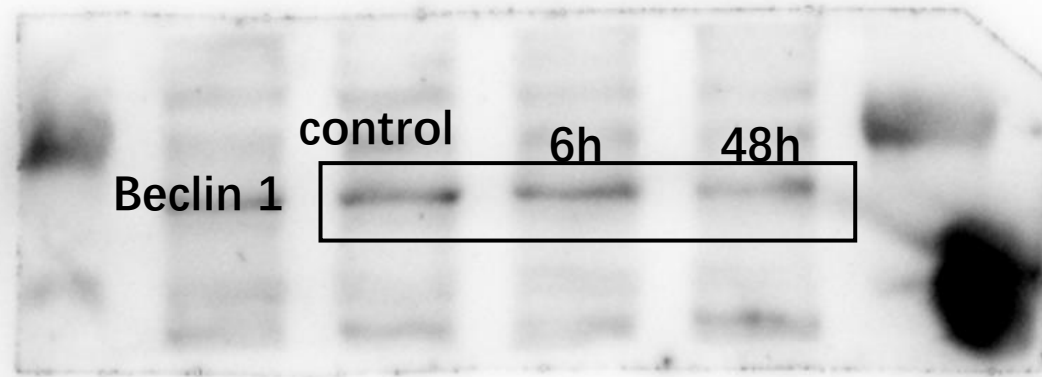

# AMPK

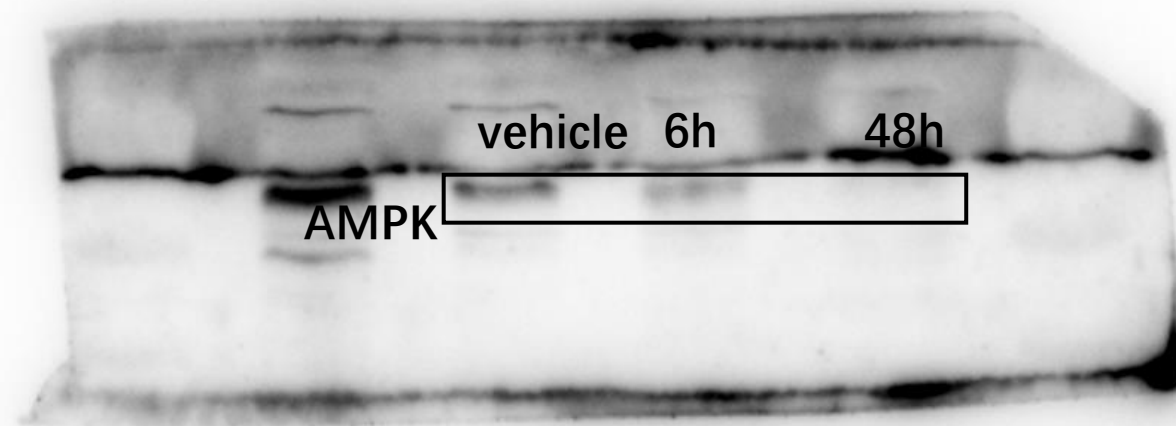

# P-AMPK

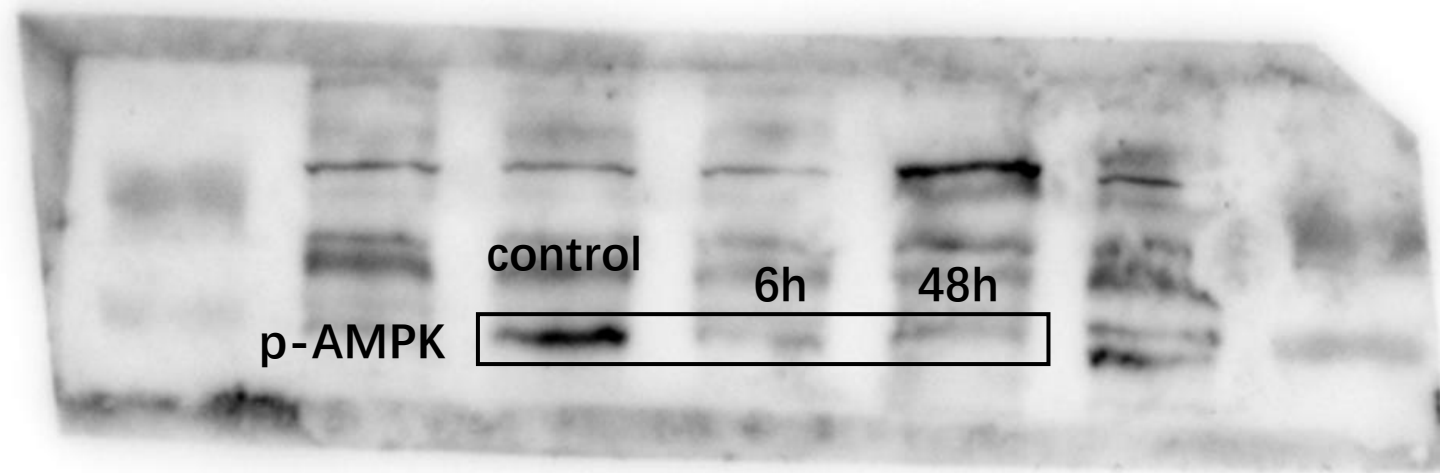

# EBP

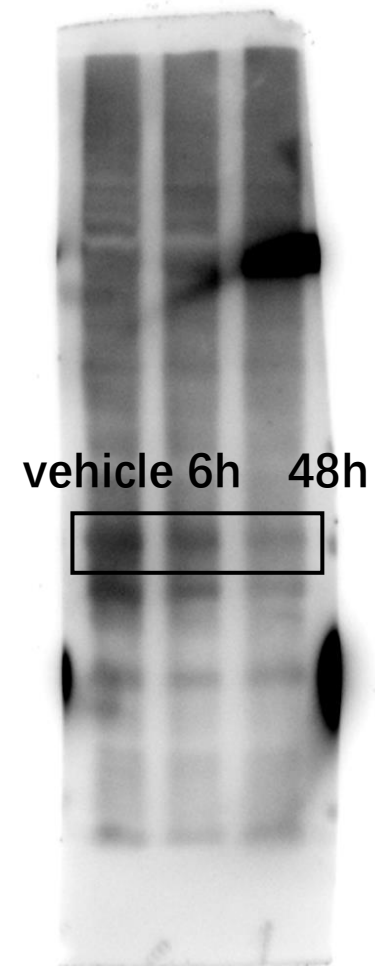

# GA

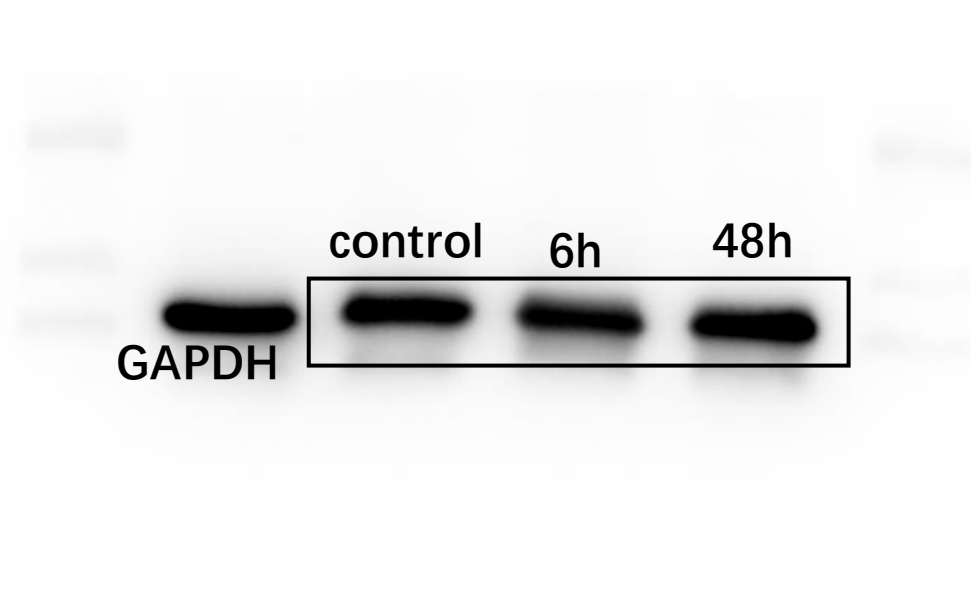

# P62-CQ

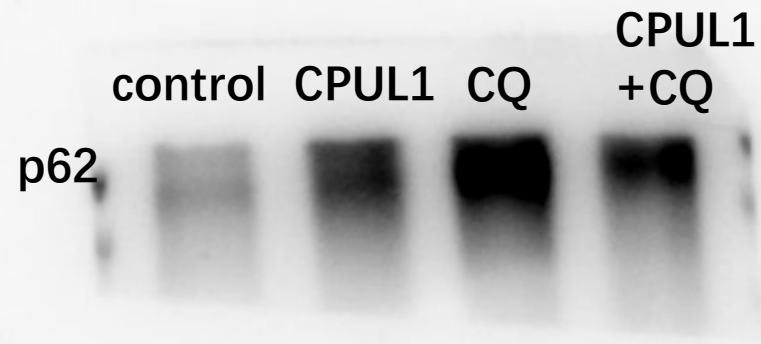

# LC3-CQ

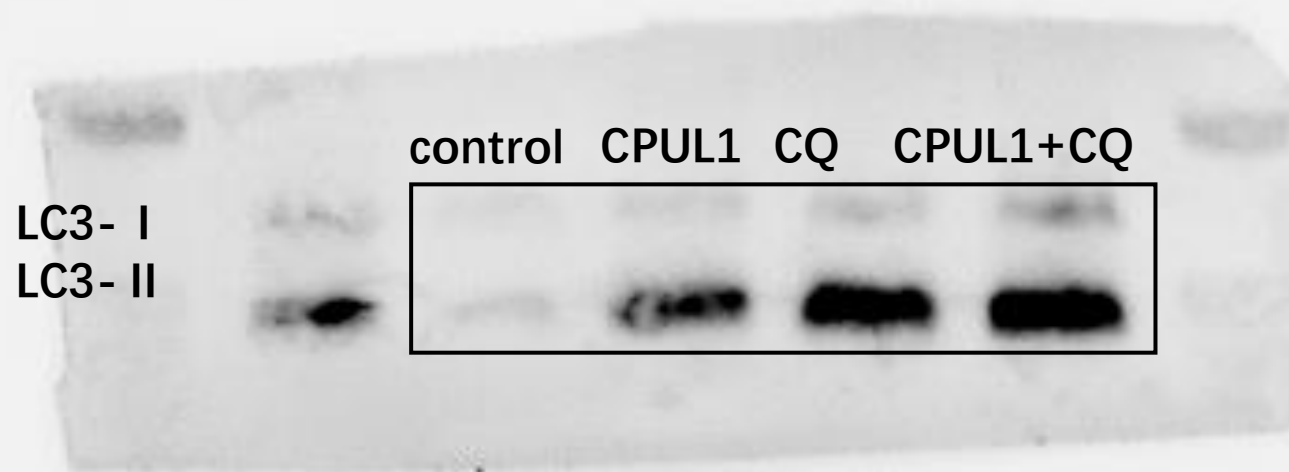

# GA-CQ

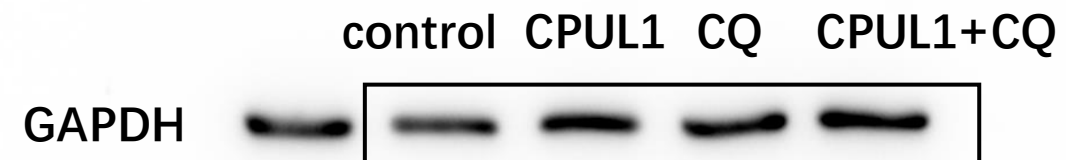

# p62-3MA

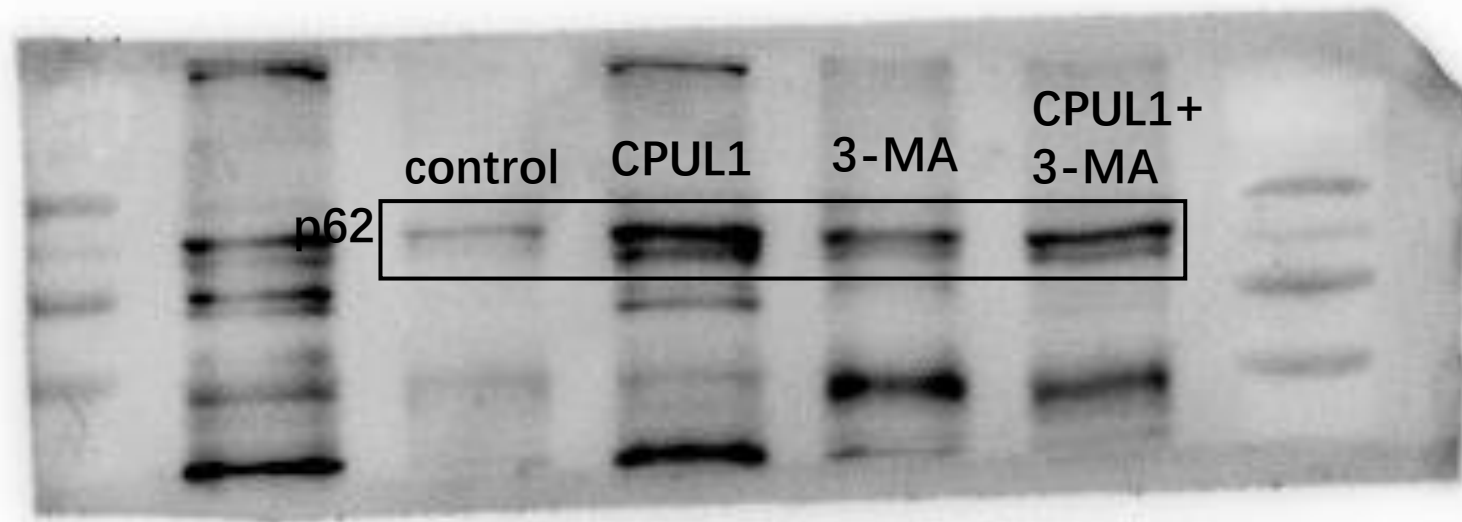

# LC3-3-MA

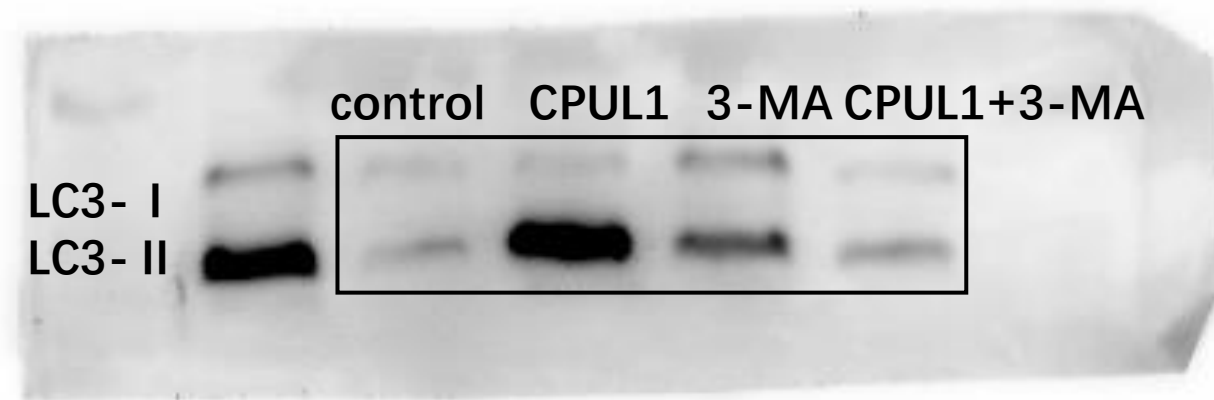

# GA-3-MA

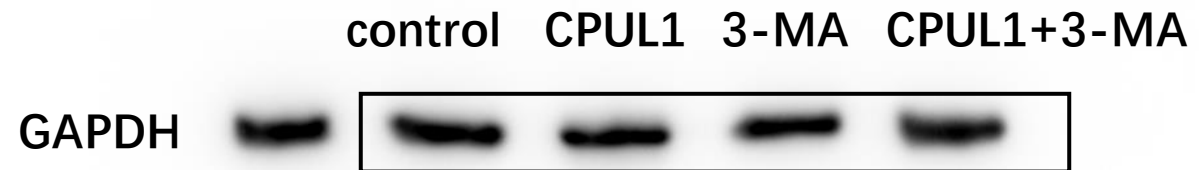

Supplement: Supplementary file 1 [file cancers-15-01607-s001.zip › Original Images for Blot.pdf]
